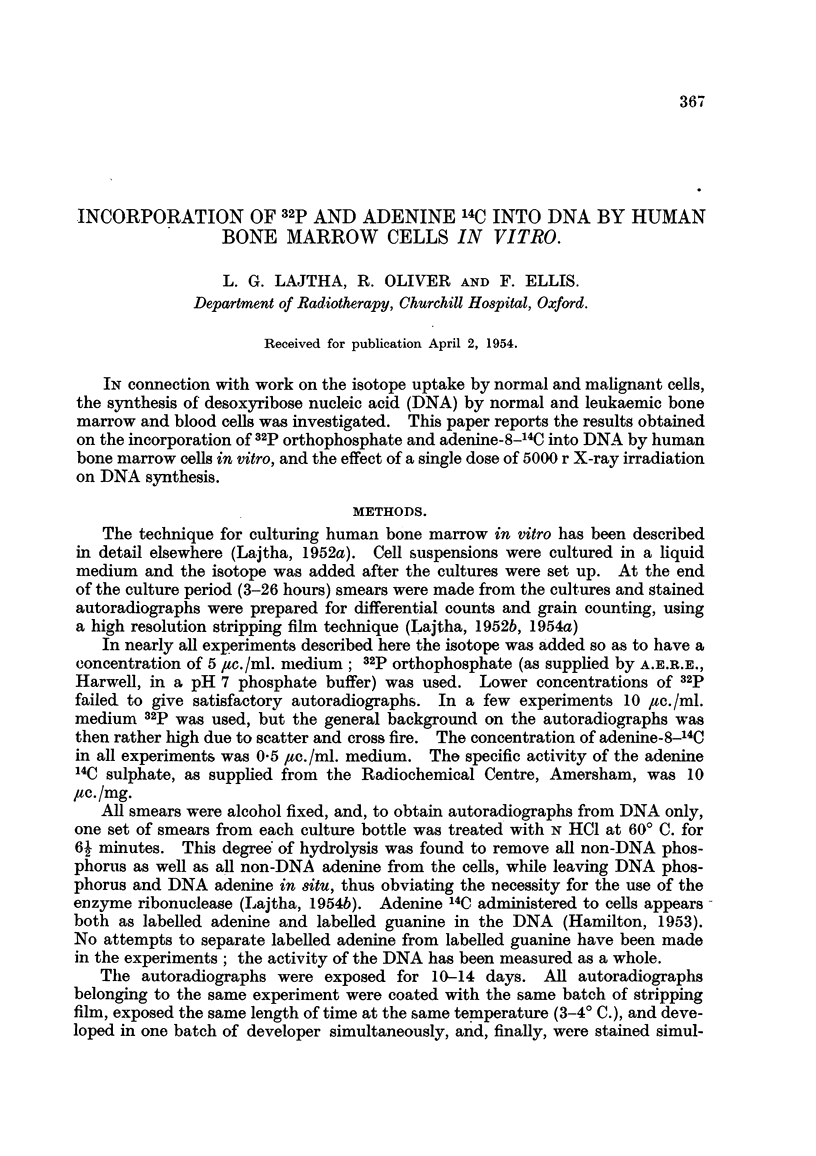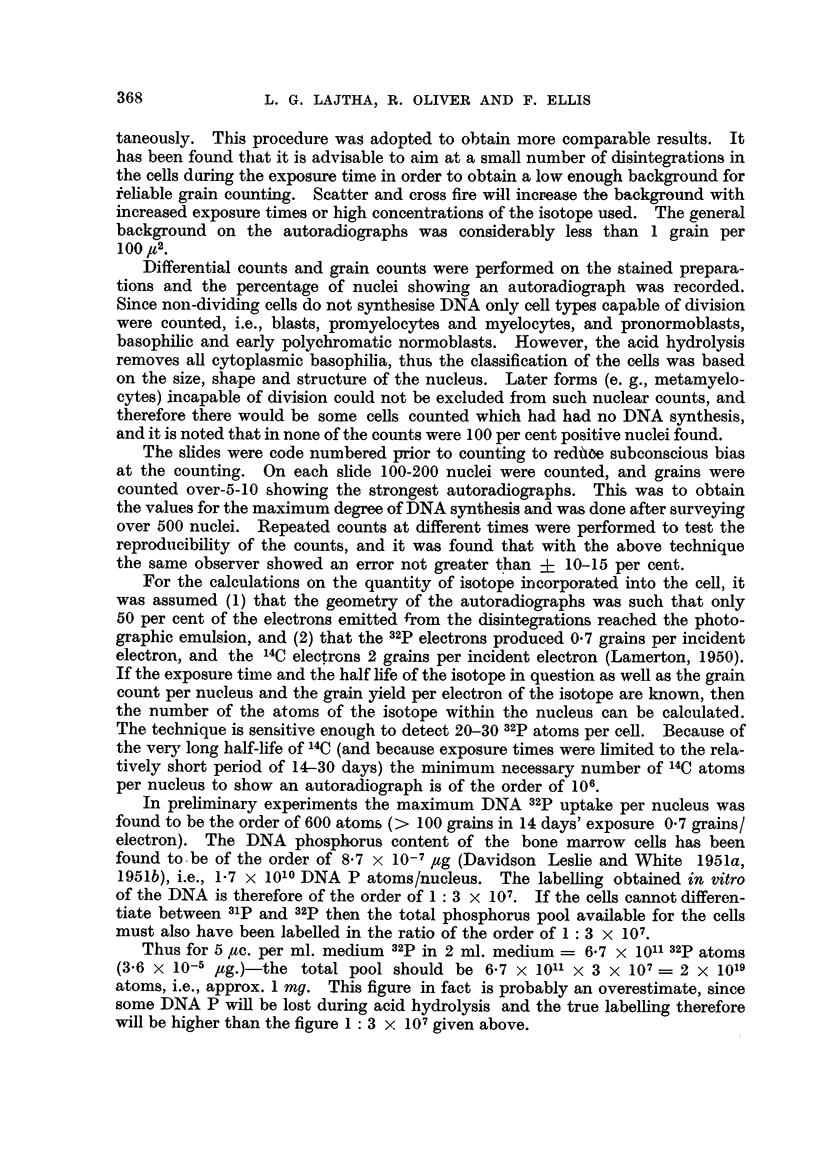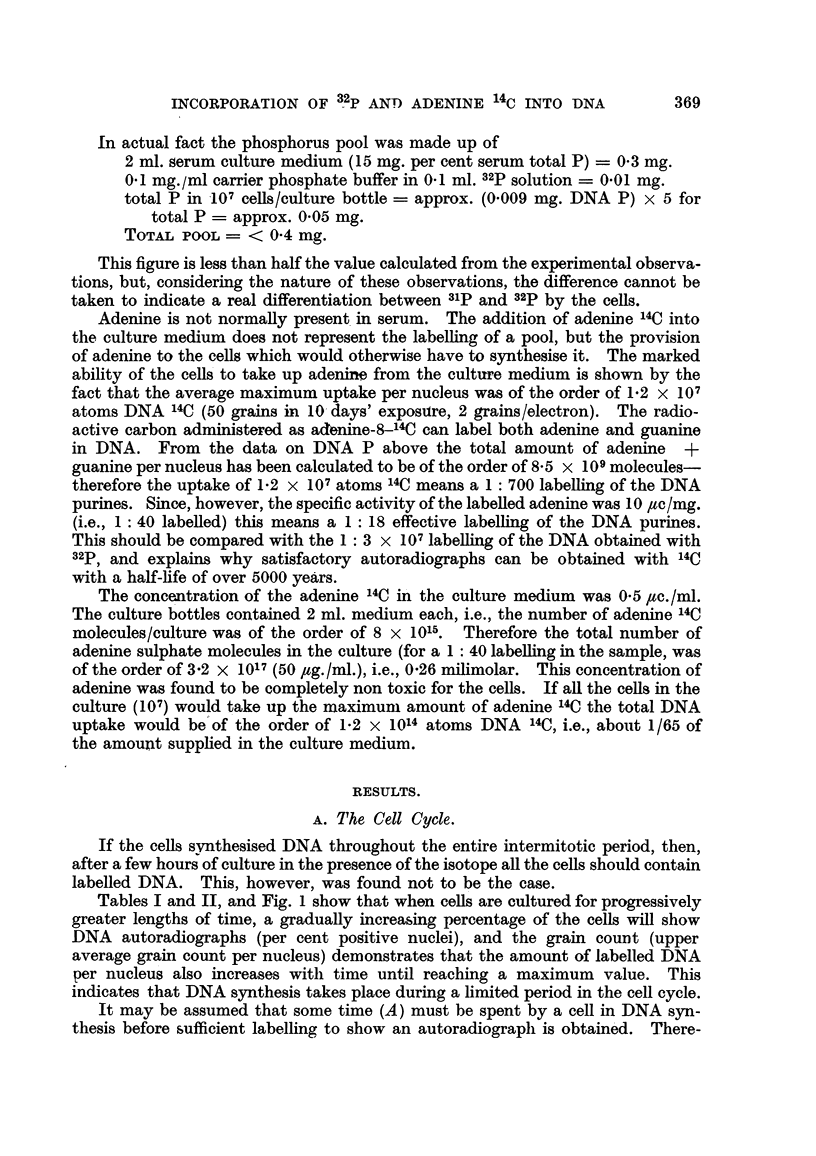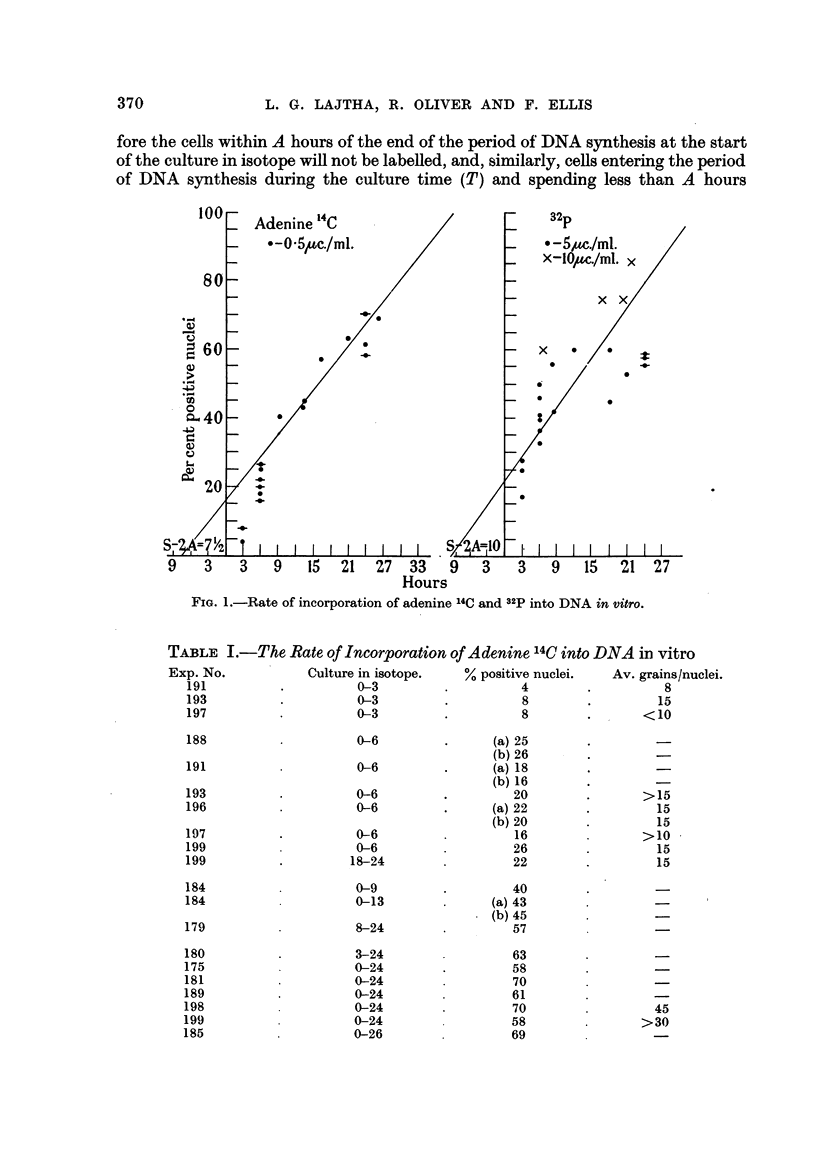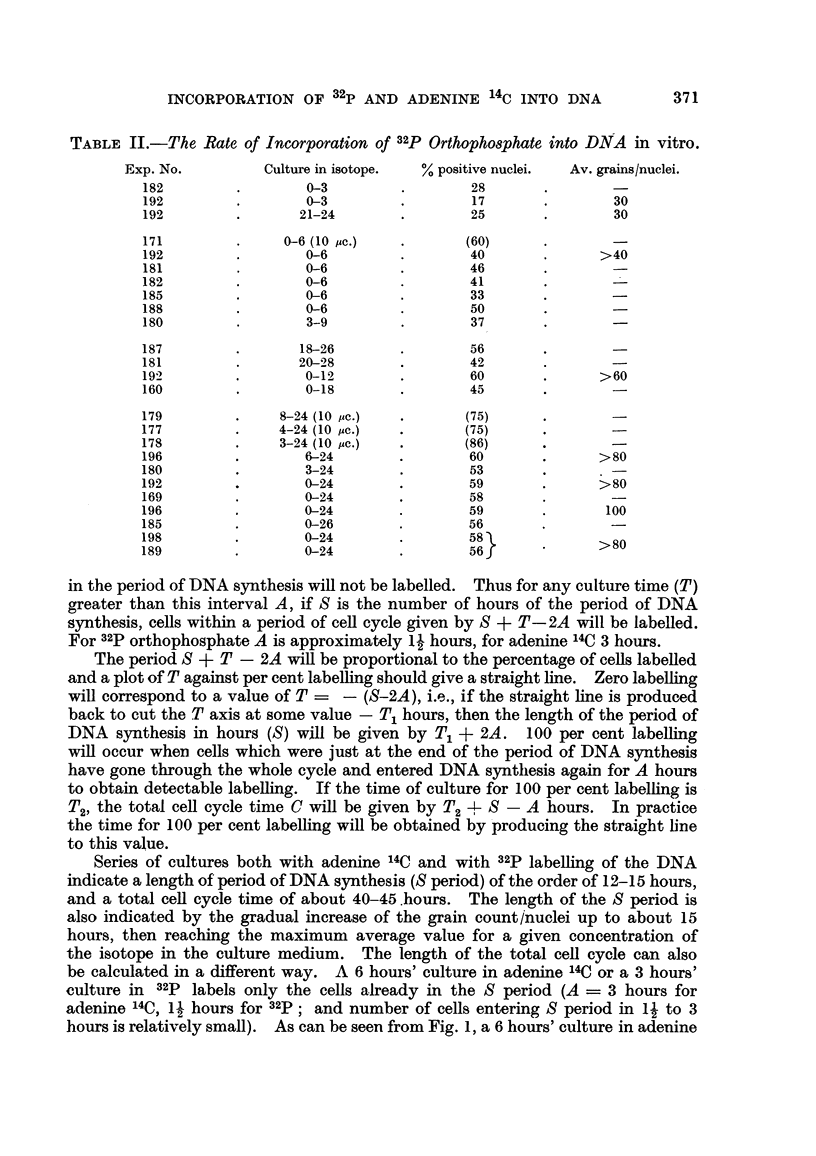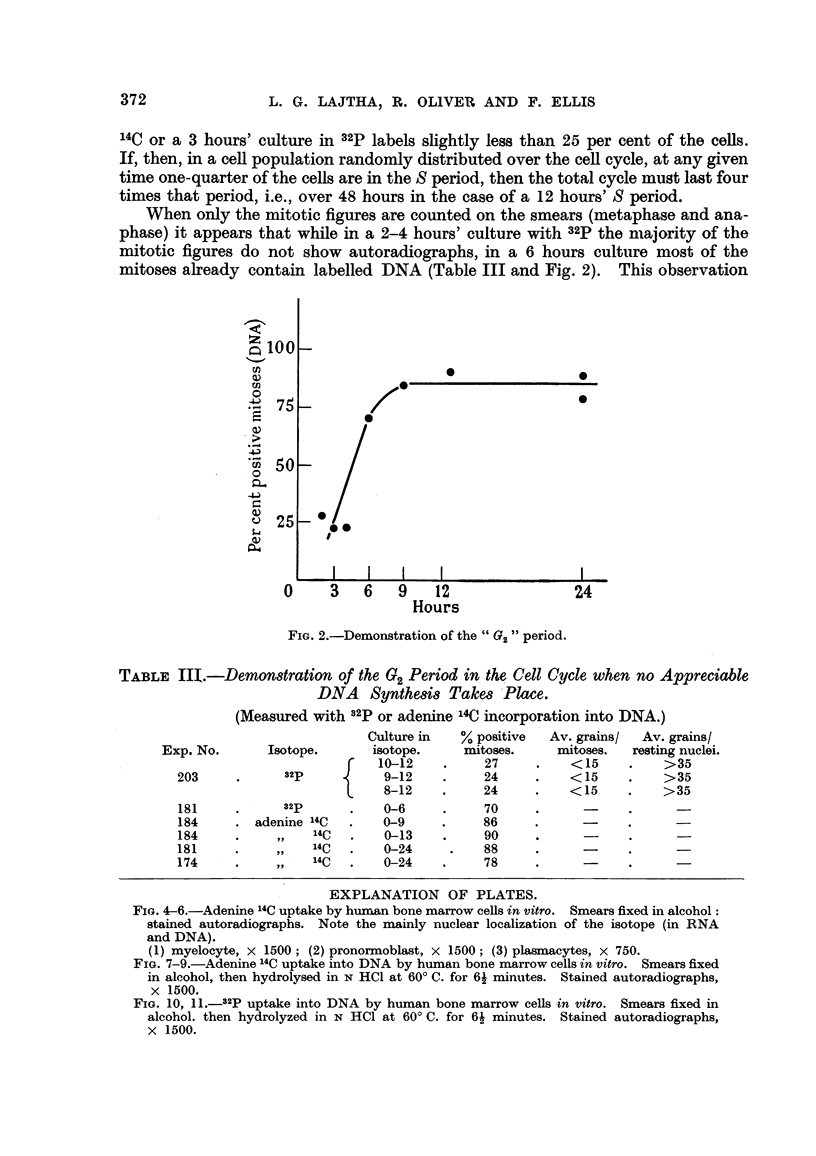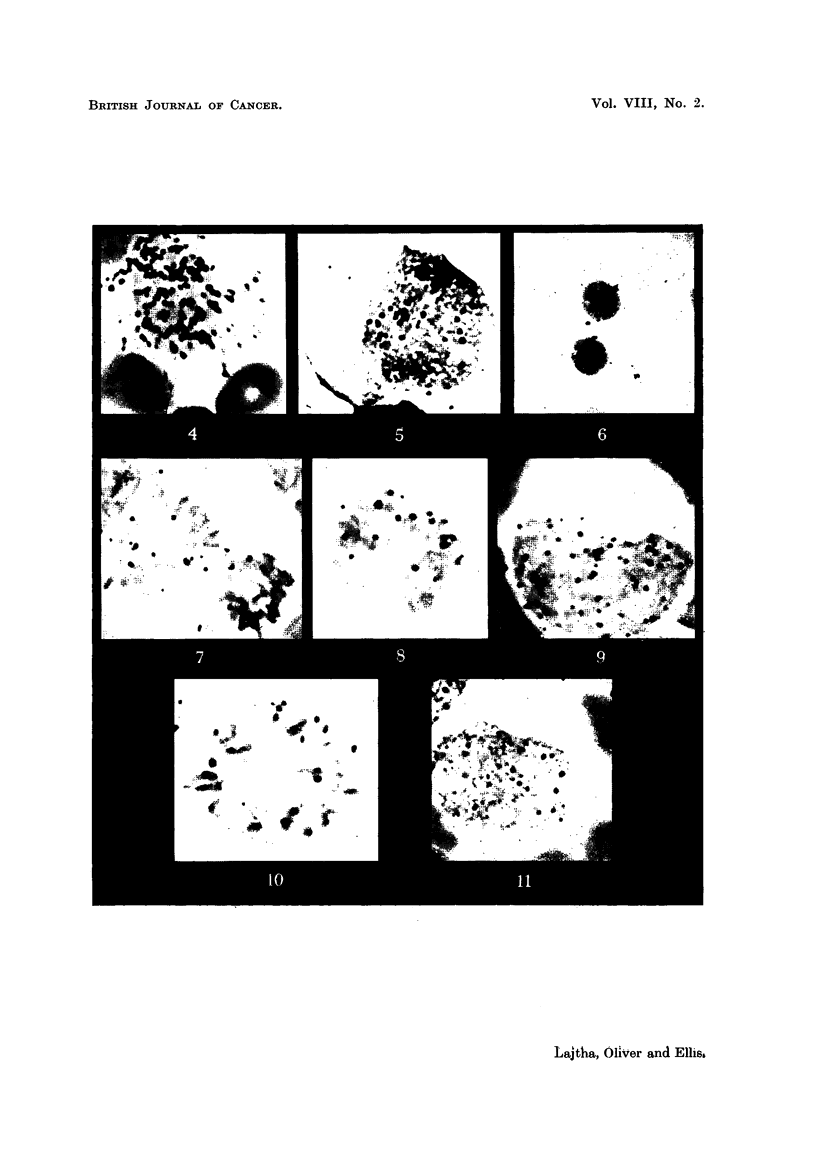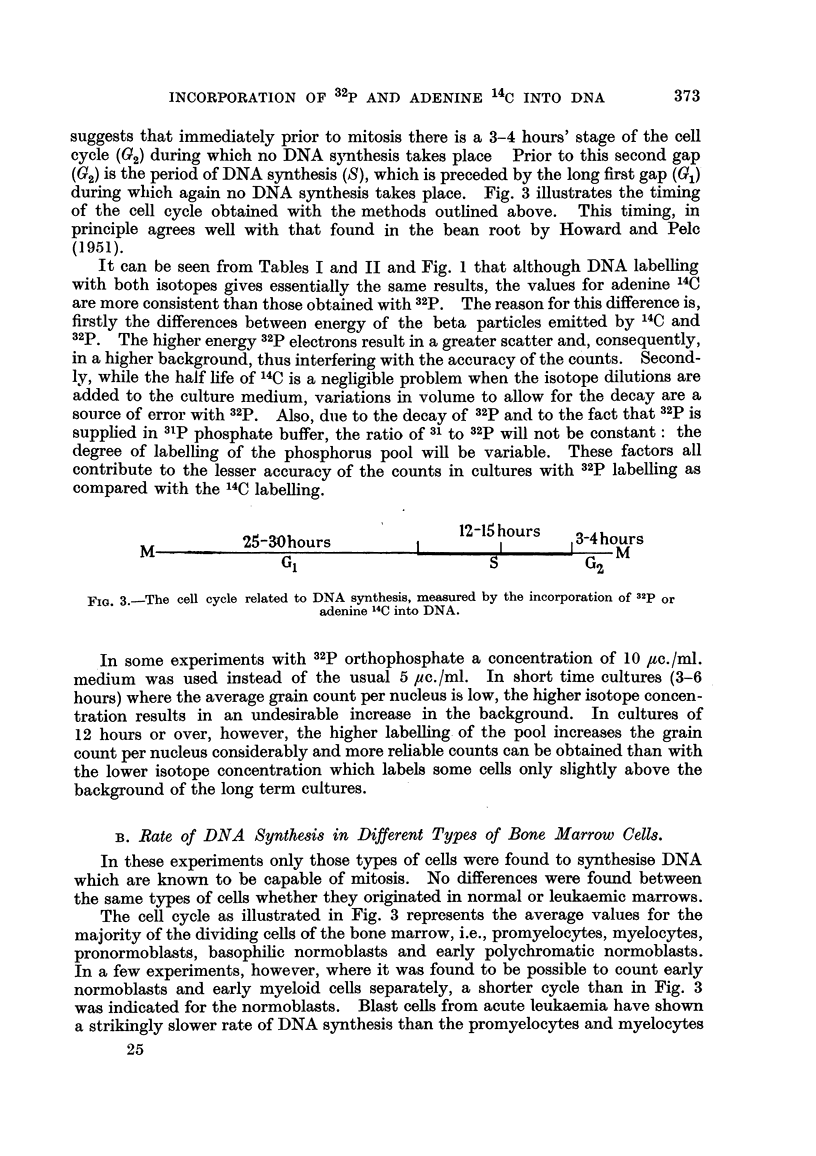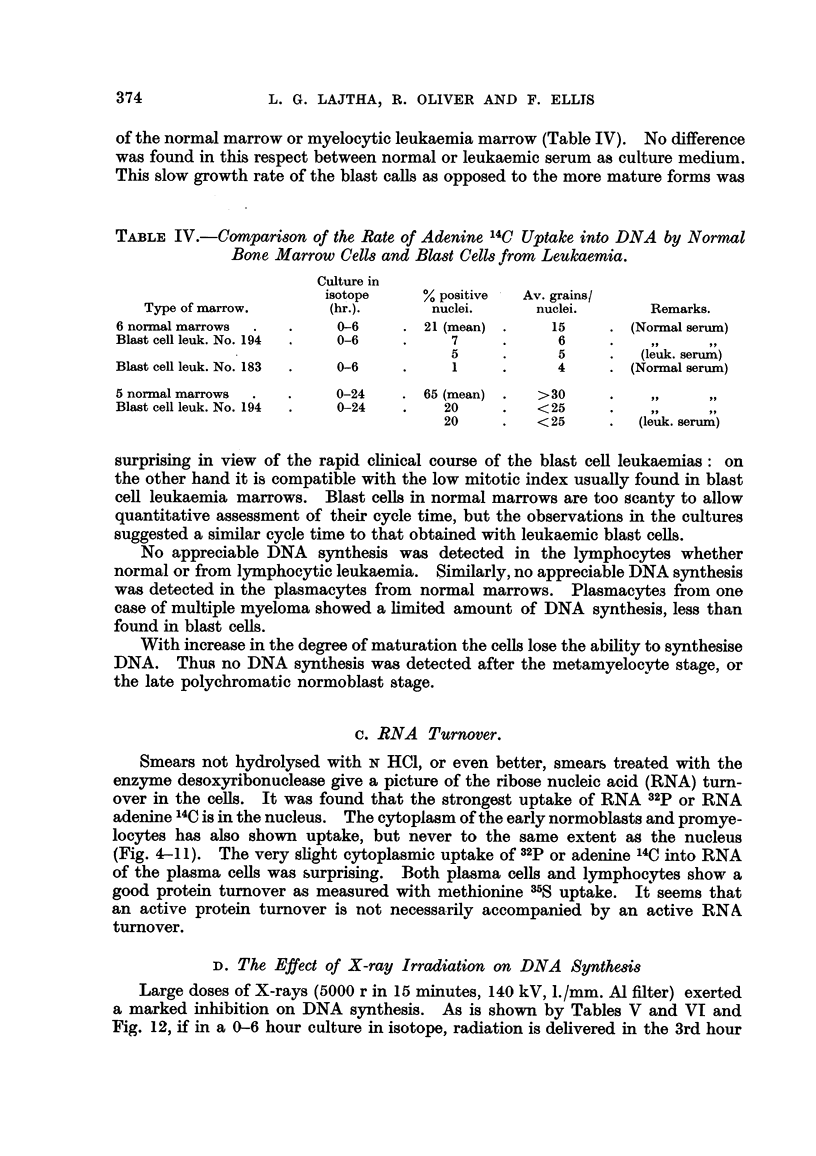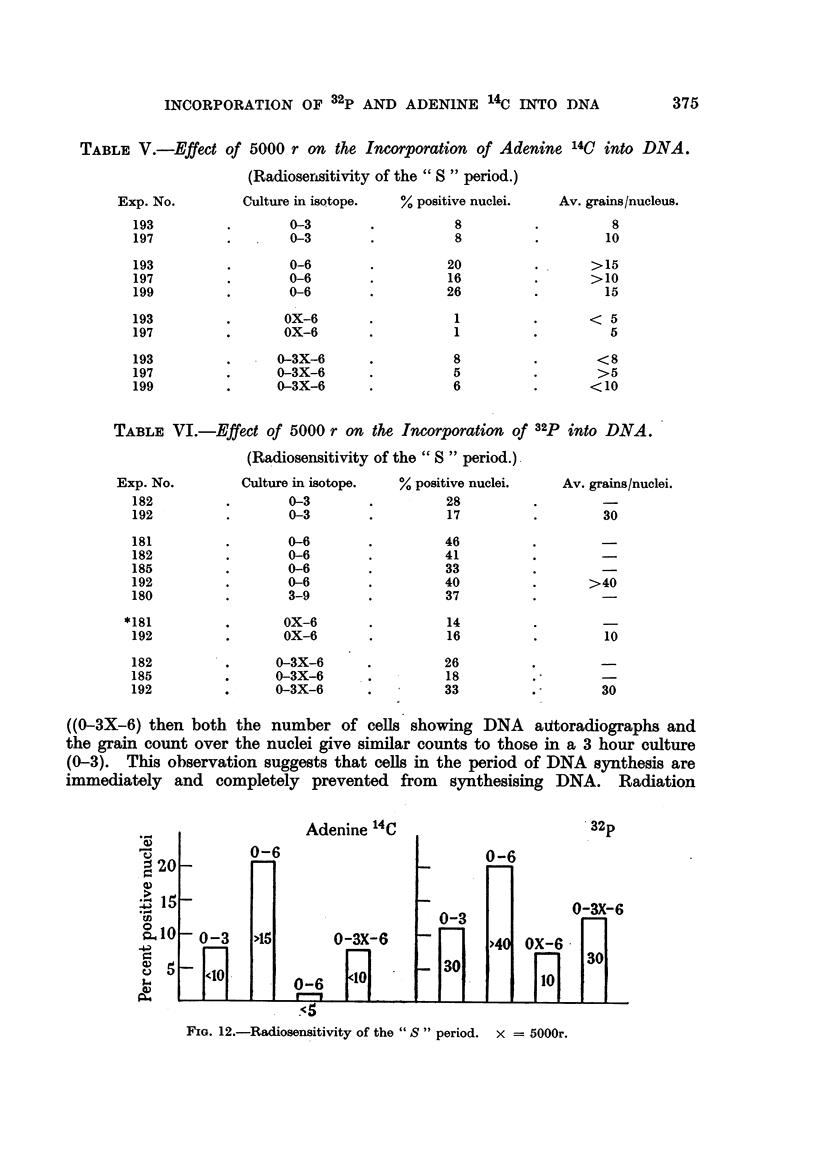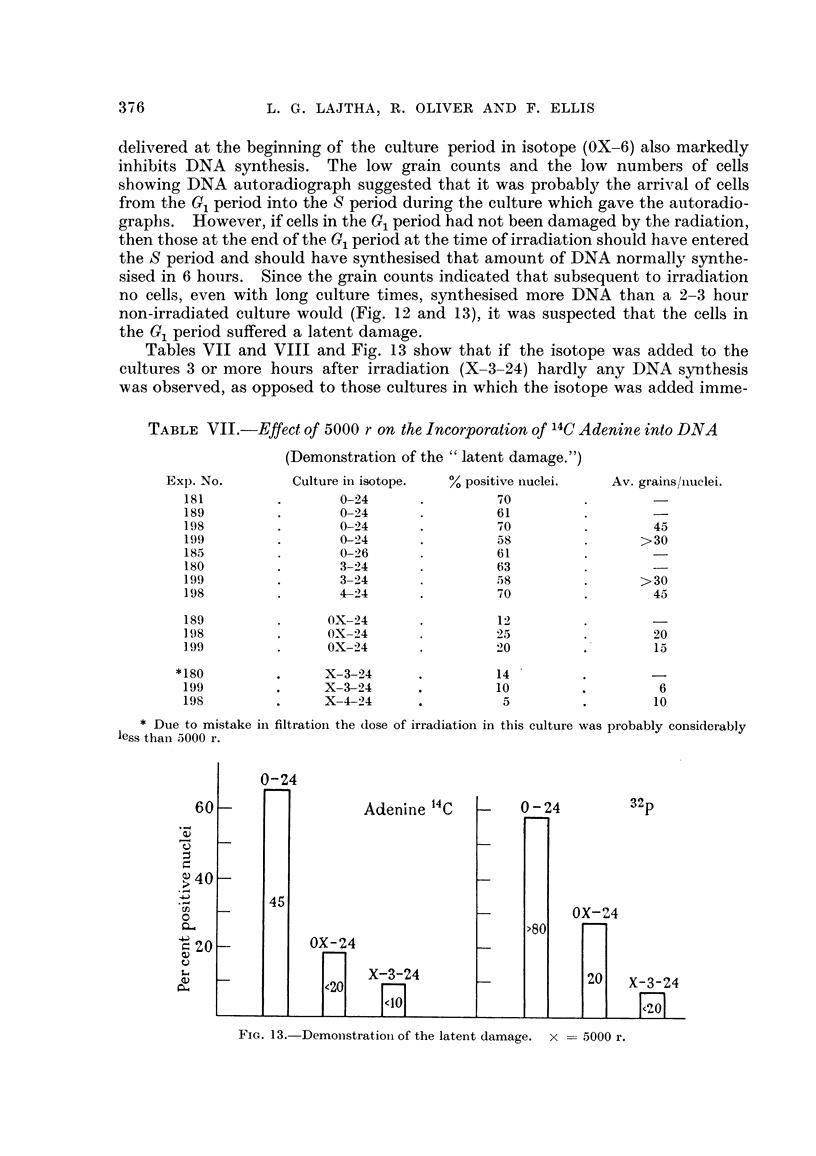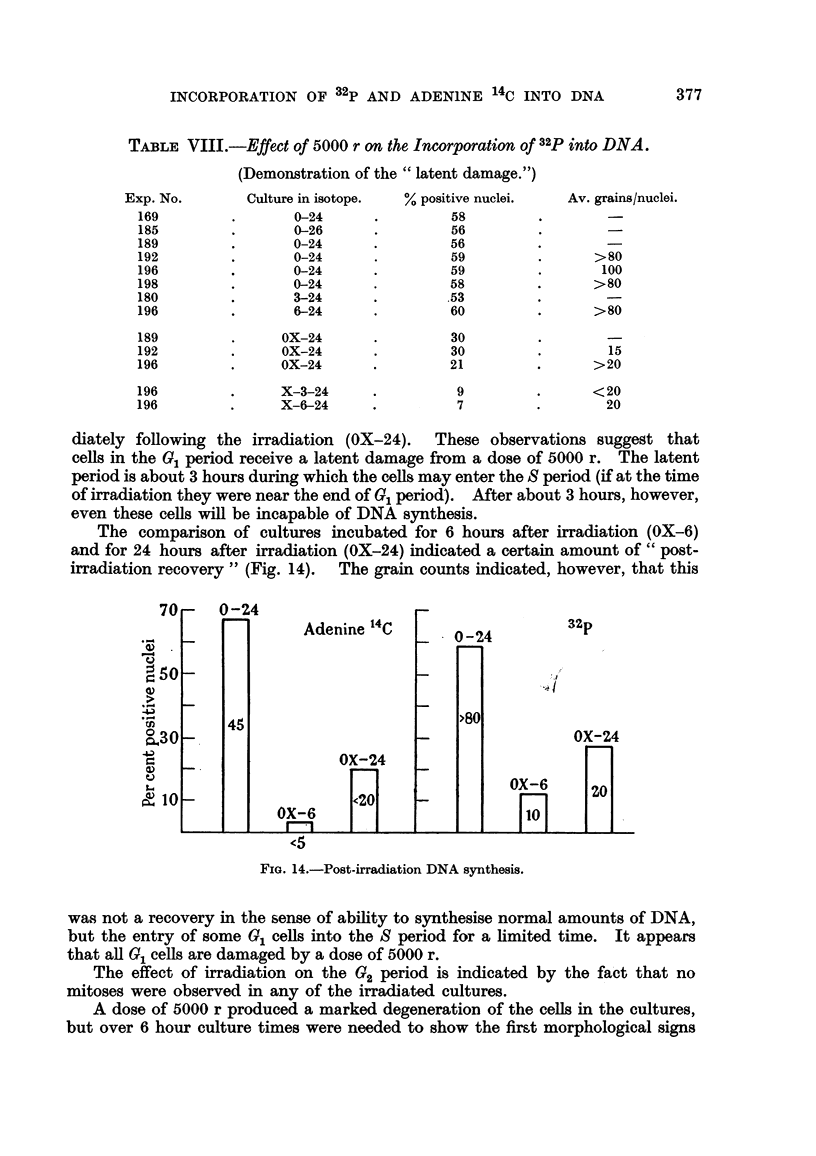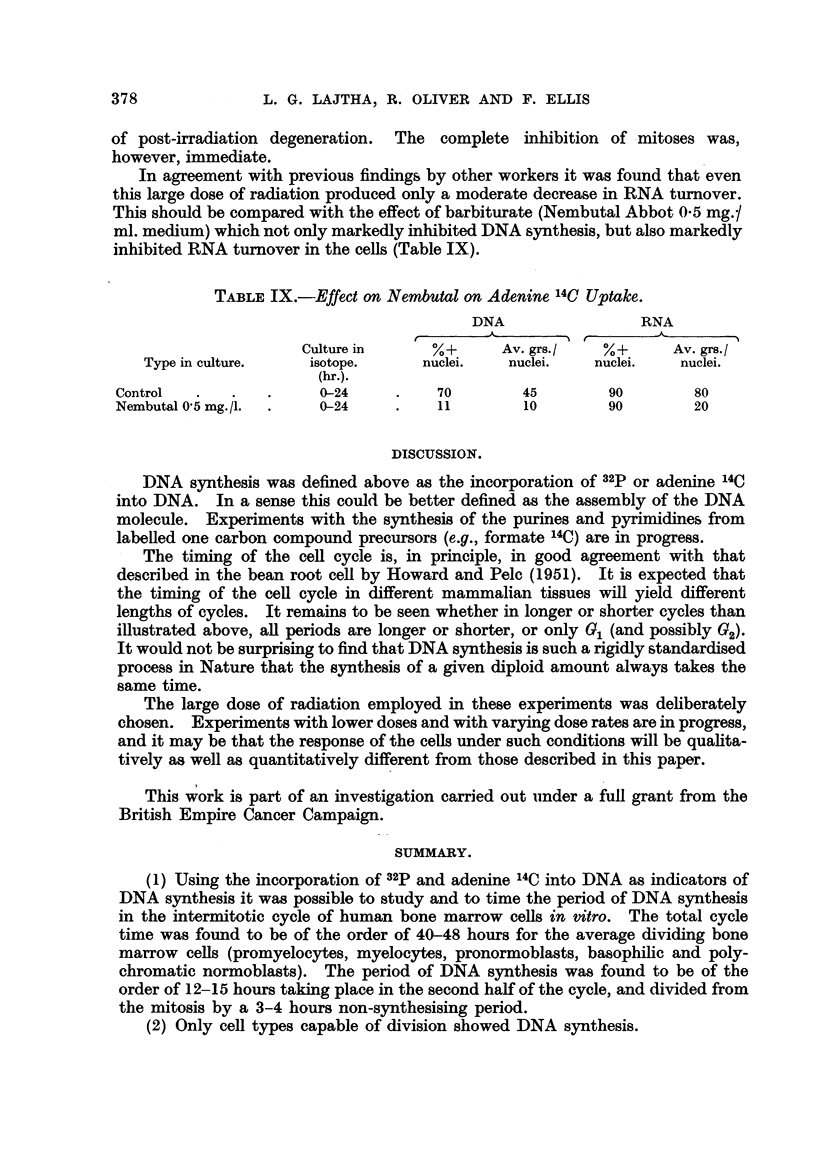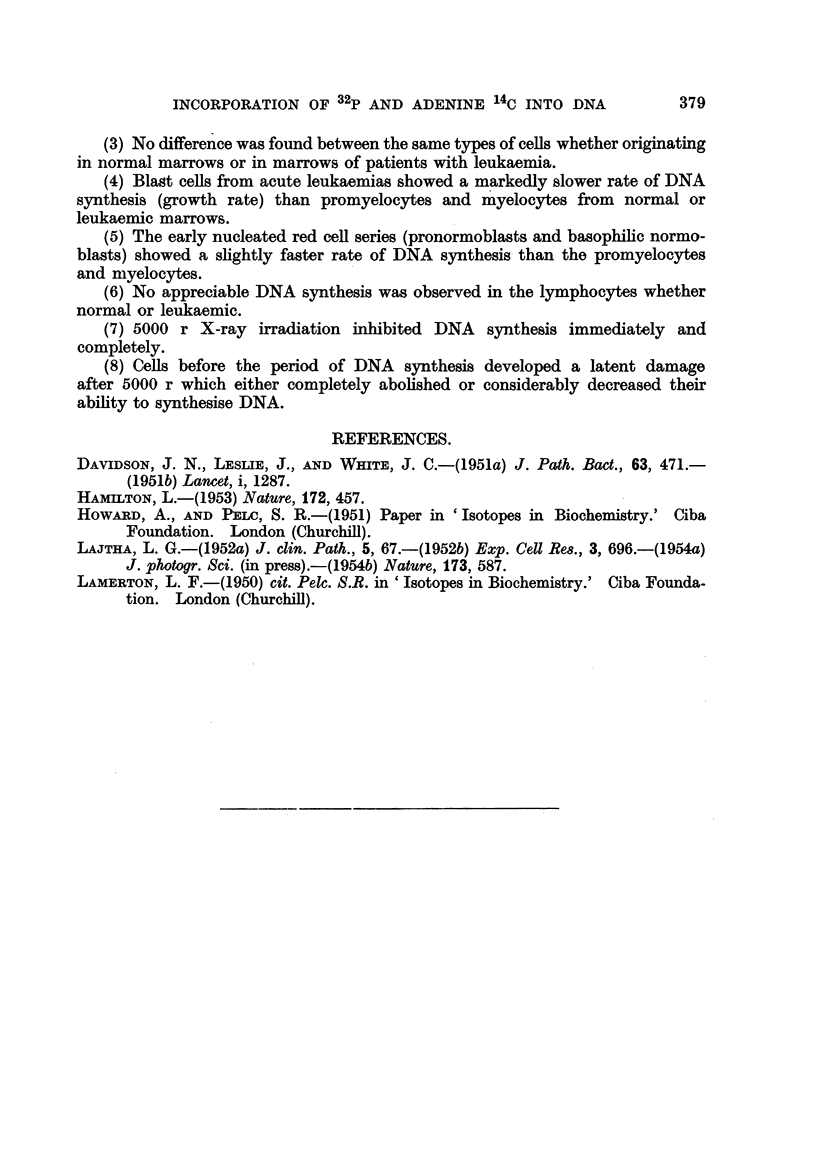# Incorporation of ^32^P and Adenine ^14^C into DNA by Human Bone Marrow Cells In Vitro

**DOI:** 10.1038/bjc.1954.38

**Published:** 1954-06

**Authors:** L. G. Lajtha, R. Oliver, F. Ellis

## Abstract

**Images:**


### INCORPORATION OF $^{32}P$ AND ADENINE $^{14}C$ INTO DNA BY HUMAN BONE MARROW CELLS *IN VITRO.*

L. G. LAJTHA, R. OLIVER AND F. ELLIS.
*Department of Radiotherapy, Churchill Hospital, Oxford.*

Received for publication April 2, 1954.

IN connection with work on the isotope uptake by normal and malignant cells, the synthesis of desoxyribose nucleic acid (DNA) by normal and leukaemic bone marrow and blood cells was investigated. This paper reports the results obtained on the incorporation of $^{32}P$ orthophosphate and adenine-8–$^{14}C$ into DNA by human bone marrow cells *in vitro*, and the effect of a single dose of 5000 r X-ray irradiation on DNA synthesis.

### METHODS.

The technique for culturing human bone marrow *in vitro* has been described in detail elsewhere (Lajtha, 1952*a*). Cell suspensions were cultured in a liquid medium and the isotope was added after the cultures were set up. At the end of the culture period (3–26 hours) smears were made from the cultures and stained autoradiographs were prepared for differential counts and grain counting, using a high resolution stripping film technique (Lajtha, 1952*b*, 1954*a*)

In nearly all experiments described here the isotope was added so as to have a concentration of 5 $\mu$c./ml. medium ; $^{32}P$ orthophosphate (as supplied by A.E.R.E., Harwell, in a pH 7 phosphate buffer) was used. Lower concentrations of $^{32}P$ failed to give satisfactory autoradiographs. In a few experiments 10 $\mu$c./ml. medium $^{32}P$ was used, but the general background on the autoradiographs was then rather high due to scatter and cross fire. The concentration of adenine-8–$^{14}C$ in all experiments was 0·5 $\mu$c./ml. medium. The specific activity of the adenine $^{14}C$ sulphate, as supplied from the Radiochemical Centre, Amersham, was 10 $\mu$c./mg.

All smears were alcohol fixed, and, to obtain autoradiographs from DNA only, one set of smears from each culture bottle was treated with N HCl at 60° C. for 6½ minutes. This degree of hydrolysis was found to remove all non-DNA phosphorus as well as all non-DNA adenine from the cells, while leaving DNA phosphorus and DNA adenine *in situ*, thus obviating the necessity for the use of the enzyme ribonuclease (Lajtha, 1954*b*). Adenine $^{14}C$ administered to cells appears both as labelled adenine and labelled guanine in the DNA (Hamilton, 1953). No attempts to separate labelled adenine from labelled guanine have been made in the experiments ; the activity of the DNA has been measured as a whole.

The autoradiographs were exposed for 10–14 days. All autoradiographs belonging to the same experiment were coated with the same batch of stripping film, exposed the same length of time at the same temperature (3–4° C.), and developed in one batch of developer simultaneously, and, finally, were stained simultaneously. This procedure was adopted to obtain more comparable results. It has been found that it is advisable to aim at a small number of disintegrations in the cells during the exposure time in order to obtain a low enough background for reliable grain counting. Scatter and cross fire will increase the background with increased exposure times or high concentrations of the isotope used. The general background on the autoradiographs was considerably less than 1 grain per 100 $\mu^2$.

Differential counts and grain counts were performed on the stained preparations and the percentage of nuclei showing an autoradiograph was recorded. Since non-dividing cells do not synthesise DNA only cell types capable of division were counted, i.e., blasts, promyelocytes and myelocytes, and pronormoblasts, basophilic and early polychromatic normoblasts. However, the acid hydrolysis removes all cytoplasmic basophilia, thus the classification of the cells was based on the size, shape and structure of the nucleus. Later forms (e. g., metamyelocytes) incapable of division could not be excluded from such nuclear counts, and therefore there would be some cells counted which had had no DNA synthesis, and it is noted that in none of the counts were 100 per cent positive nuclei found.

The slides were code numbered prior to counting to reduce subconscious bias at the counting. On each slide 100-200 nuclei were counted, and grains were counted over-5-10 showing the strongest autoradiographs. This was to obtain the values for the maximum degree of DNA synthesis and was done after surveying over 500 nuclei. Repeated counts at different times were performed to test the reproducibility of the counts, and it was found that with the above technique the same observer showed an error not greater than $\pm$ 10–15 per cent.

For the calculations on the quantity of isotope incorporated into the cell, it was assumed (1) that the geometry of the autoradiographs was such that only 50 per cent of the electrons emitted from the disintegrations reached the photographic emulsion, and (2) that the $^{32}$P electrons produced 0·7 grains per incident electron, and the $^{14}$C electrons 2 grains per incident electron (Lamerton, 1950). If the exposure time and the half life of the isotope in question as well as the grain count per nucleus and the grain yield per electron of the isotope are known, then the number of the atoms of the isotope within the nucleus can be calculated. The technique is sensitive enough to detect 20–30 $^{32}$P atoms per cell. Because of the very long half-life of $^{14}$C (and because exposure times were limited to the relatively short period of 14–30 days) the minimum necessary number of $^{14}$C atoms per nucleus to show an autoradiograph is of the order of $10^6$.

In preliminary experiments the maximum DNA $^{32}$P uptake per nucleus was found to be the order of 600 atoms ($>$ 100 grains in 14 days' exposure 0·7 grains/electron). The DNA phosphorus content of the bone marrow cells has been found to be of the order of $8{\cdot}7 \times 10^{-7}$ $\mu$g (Davidson Leslie and White 1951*a*, 1951*b*), i.e., $1{\cdot}7 \times 10^{10}$ DNA P atoms/nucleus. The labelling obtained *in vitro* of the DNA is therefore of the order of $1 : 3 \times 10^7$. If the cells cannot differentiate between $^{31}$P and $^{32}$P then the total phosphorus pool available for the cells must also have been labelled in the ratio of the order of $1 : 3 \times 10^7$.

Thus for 5 $\mu$c. per ml. medium $^{32}$P in 2 ml. medium $= 6{\cdot}7 \times 10^{11}$ $^{32}$P atoms ($3{\cdot}6 \times 10^{-5}$ $\mu$g.)—the total pool should be $6{\cdot}7 \times 10^{11} \times 3 \times 10^7 = 2 \times 10^{19}$ atoms, i.e., approx. 1 *mg*. This figure in fact is probably an overestimate, since some DNA P will be lost during acid hydrolysis and the true labelling therefore will be higher than the figure $1 : 3 \times 10^7$ given above.

In actual fact the phosphorus pool was made up of
2 ml. serum culture medium (15 mg. per cent serum total P) = 0·3 mg.
0·1 mg./ml carrier phosphate buffer in 0·1 ml. $^{32}P$ solution = 0·01 mg.
total P in $10^7$ cells/culture bottle = approx. (0·009 mg. DNA P) $\times$ 5 for total P = approx. 0·05 mg.
TOTAL POOL = $<$ 0·4 mg.

This figure is less than half the value calculated from the experimental observations, but, considering the nature of these observations, the difference cannot be taken to indicate a real differentiation between $^{31}P$ and $^{32}P$ by the cells.

Adenine is not normally present in serum. The addition of adenine $^{14}C$ into the culture medium does not represent the labelling of a pool, but the provision of adenine to the cells which would otherwise have to synthesise it. The marked ability of the cells to take up adenine from the culture medium is shown by the fact that the average maximum uptake per nucleus was of the order of $1{\cdot}2 \times 10^7$ atoms DNA $^{14}C$ (50 grains in 10 days' exposure, 2 grains/electron). The radioactive carbon administered as adenine-8–$^{14}C$ can label both adenine and guanine in DNA. From the data on DNA P above the total amount of adenine + guanine per nucleus has been calculated to be of the order of $8{\cdot}5 \times 10^9$ molecules—therefore the uptake of $1{\cdot}2 \times 10^7$ atoms $^{14}C$ means a 1 : 700 labelling of the DNA purines. Since, however, the specific activity of the labelled adenine was 10 $\mu$c/mg. (i.e., 1 : 40 labelled) this means a 1 : 18 effective labelling of the DNA purines. This should be compared with the 1 : 3 $\times 10^7$ labelling of the DNA obtained with $^{32}P$, and explains why satisfactory autoradiographs can be obtained with $^{14}C$ with a half-life of over 5000 years.

The concentration of the adenine $^{14}C$ in the culture medium was 0·5 $\mu$c./ml. The culture bottles contained 2 ml. medium each, i.e., the number of adenine $^{14}C$ molecules/culture was of the order of $8 \times 10^{15}$. Therefore the total number of adenine sulphate molecules in the culture (for a 1 : 40 labelling in the sample, was of the order of $3{\cdot}2 \times 10^{17}$ (50 $\mu$g./ml.), i.e., 0·26 milimolar. This concentration of adenine was found to be completely non toxic for the cells. If all the cells in the culture ($10^7$) would take up the maximum amount of adenine $^{14}C$ the total DNA uptake would be of the order of $1{\cdot}2 \times 10^{14}$ atoms DNA $^{14}C$, i.e., about 1/65 of the amount supplied in the culture medium.

### RESULTS.

### A. *The Cell Cycle.*

If the cells synthesised DNA throughout the entire intermitotic period, then, after a few hours of culture in the presence of the isotope all the cells should contain labelled DNA. This, however, was found not to be the case.

Tables I and II, and Fig. 1 show that when cells are cultured for progressively greater lengths of time, a gradually increasing percentage of the cells will show DNA autoradiographs (per cent positive nuclei), and the grain count (upper average grain count per nucleus) demonstrates that the amount of labelled DNA per nucleus also increases with time until reaching a maximum value. This indicates that DNA synthesis takes place during a limited period in the cell cycle.

It may be assumed that some time ($A$) must be spent by a cell in DNA synthesis before sufficient labelling to show an autoradiograph is obtained. Therefore the cells within $A$ hours of the end of the period of DNA synthesis at the start of the culture in isotope will not be labelled, and, similarly, cells entering the period of DNA synthesis during the culture time ($T$) and spending less than $A$ hours

FIG. 1.—Rate of incorporation of adenine $^{14}C$ and $^{32}P$ into DNA *in vitro.*

TABLE I.—*The Rate of Incorporation of Adenine* $^{14}C$ *into DNA* in vitro

| Exp. No. | Culture in isotope. | % positive nuclei. | Av. grains/nuclei. |
|---|---|---|---|
| 191 | 0–3 | 4 | 8 |
| 193 | 0–3 | 8 | 15 |
| 197 | 0–3 | 8 | <10 |
| 188 | 0–6 | (a) 25 | — |
| | | (b) 26 | — |
| 191 | 0–6 | (a) 18 | — |
| | | (b) 16 | — |
| 193 | 0–6 | 20 | >15 |
| 196 | 0–6 | (a) 22 | 15 |
| | | (b) 20 | 15 |
| 197 | 0–6 | 16 | >10 |
| 199 | 0–6 | 26 | 15 |
| 199 | 18–24 | 22 | 15 |
| 184 | 0–9 | 40 | — |
| 184 | 0–13 | (a) 43 | — |
| | | (b) 45 | — |
| 179 | 8–24 | 57 | — |
| 180 | 3–24 | 63 | — |
| 175 | 0–24 | 58 | — |
| 181 | 0–24 | 70 | — |
| 189 | 0–24 | 61 | — |
| 198 | 0–24 | 70 | 45 |
| 199 | 0–24 | 58 | >30 |
| 185 | 0–26 | 69 | — |

TABLE II.—*The Rate of Incorporation of $^{32}$P Orthophosphate into DNA* in vitro.

| Exp. No. | Culture in isotope. | % positive nuclei. | Av. grains/nuclei. |
|---|---|---|---|
| 182 | 0–3 | 28 | — |
| 192 | 0–3 | 17 | 30 |
| 192 | 21–24 | 25 | 30 |
| 171 | 0–6 (10 μc.) | (60) | — |
| 192 | 0–6 | 40 | >40 |
| 181 | 0–6 | 46 | — |
| 182 | 0–6 | 41 | — |
| 185 | 0–6 | 33 | — |
| 188 | 0–6 | 50 | — |
| 180 | 3–9 | 37 | — |
| 187 | 18–26 | 56 | — |
| 181 | 20–28 | 42 | — |
| 192 | 0–12 | 60 | >60 |
| 160 | 0–18 | 45 | — |
| 179 | 8–24 (10 μc.) | (75) | — |
| 177 | 4–24 (10 μc.) | (75) | — |
| 178 | 3–24 (10 μc.) | (86) | — |
| 196 | 6–24 | 60 | >80 |
| 180 | 3–24 | 53 | — |
| 192 | 0–24 | 59 | >80 |
| 169 | 0–24 | 58 | — |
| 196 | 0–24 | 59 | 100 |
| 185 | 0–26 | 56 | — |
| 198 | 0–24 | 58 } | >80 |
| 189 | 0–24 | 56 } | |

in the period of DNA synthesis will not be labelled. Thus for any culture time ($T$) greater than this interval $A$, if $S$ is the number of hours of the period of DNA synthesis, cells within a period of cell cycle given by $S + T - 2A$ will be labelled. For $^{32}$P orthophosphate $A$ is approximately $1\frac{1}{2}$ hours, for adenine $^{14}$C 3 hours.

The period $S + T - 2A$ will be proportional to the percentage of cells labelled and a plot of $T$ against per cent labelling should give a straight line. Zero labelling will correspond to a value of $T = -(S-2A)$, i.e., if the straight line is produced back to cut the $T$ axis at some value $-T_1$ hours, then the length of the period of DNA synthesis in hours ($S$) will be given by $T_1 + 2A$. 100 per cent labelling will occur when cells which were just at the end of the period of DNA synthesis have gone through the whole cycle and entered DNA synthesis again for $A$ hours to obtain detectable labelling. If the time of culture for 100 per cent labelling is $T_2$, the total cell cycle time $C$ will be given by $T_2 + S - A$ hours. In practice the time for 100 per cent labelling will be obtained by producing the straight line to this value.

Series of cultures both with adenine $^{14}$C and with $^{32}$P labelling of the DNA indicate a length of period of DNA synthesis ($S$ period) of the order of 12–15 hours, and a total cell cycle time of about 40–45 hours. The length of the $S$ period is also indicated by the gradual increase of the grain count/nuclei up to about 15 hours, then reaching the maximum average value for a given concentration of the isotope in the culture medium. The length of the total cell cycle can also be calculated in a different way. A 6 hours' culture in adenine $^{14}$C or a 3 hours' culture in $^{32}$P labels only the cells already in the $S$ period ($A = 3$ hours for adenine $^{14}$C, $1\frac{1}{2}$ hours for $^{32}$P ; and number of cells entering $S$ period in $1\frac{1}{2}$ to 3 hours is relatively small). As can be seen from Fig. 1, a 6 hours' culture in adenine

$^{14}C$ or a 3 hours' culture in $^{32}P$ labels slightly less than 25 per cent of the cells. If, then, in a cell population randomly distributed over the cell cycle, at any given time one-quarter of the cells are in the *S* period, then the total cycle must last four times that period, i.e., over 48 hours in the case of a 12 hours' *S* period.

When only the mitotic figures are counted on the smears (metaphase and anaphase) it appears that while in a 2–4 hours' culture with $^{32}P$ the majority of the mitotic figures do not show autoradiographs, in a 6 hours culture most of the mitoses already contain labelled DNA (Table III and Fig. 2). This observation

FIG. 2.—Demonstration of the " $G_2$ " period.

TABLE III.—*Demonstration of the $G_2$ Period in the Cell Cycle when no Appreciable DNA Synthesis Takes Place.*

(Measured with $^{32}P$ or adenine $^{14}C$ incorporation into DNA.)

| Exp. No. | Isotope. | Culture in isotope. | % positive mitoses. | Av. grains/ mitoses. | Av. grains/ resting nuclei. |
|---|---|---|---|---|---|
| 203 | $^{32}P$ | 10–12 | 27 | <15 | >35 |
| | | 9–12 | 24 | <15 | >35 |
| | | 8–12 | 24 | <15 | >35 |
| 181 | $^{32}P$ | 0–6 | 70 | — | — |
| 184 | adenine $^{14}C$ | 0–9 | 86 | — | — |
| 184 | " $^{14}C$ | 0–13 | 90 | — | — |
| 181 | " $^{14}C$ | 0–24 | 88 | — | — |
| 174 | " $^{14}C$ | 0–24 | 78 | — | — |

EXPLANATION OF PLATES.

FIG. 4–6.—Adenine $^{14}C$ uptake by human bone marrow cells *in vitro*. Smears fixed in alcohol: stained autoradiographs. Note the mainly nuclear localization of the isotope (in RNA and DNA).

(1) myelocyte, × 1500; (2) pronormoblast, × 1500; (3) plasmacytes, × 750.

FIG. 7–9.—Adenine $^{14}C$ uptake into DNA by human bone marrow cells *in vitro*. Smears fixed in alcohol, then hydrolysed in N HCl at 60° C. for 6½ minutes. Stained autoradiographs, × 1500.

FIG. 10, 11.—$^{32}P$ uptake into DNA by human bone marrow cells *in vitro*. Smears fixed in alcohol. then hydrolyzed in N HCl at 60° C. for 6½ minutes. Stained autoradiographs, × 1500.

Lajtha, Oliver and Ellis.

suggests that immediately prior to mitosis there is a 3–4 hours' stage of the cell cycle ($G_2$) during which no DNA synthesis takes place Prior to this second gap ($G_2$) is the period of DNA synthesis ($S$), which is preceded by the long first gap ($G_1$) during which again no DNA synthesis takes place. Fig. 3 illustrates the timing of the cell cycle obtained with the methods outlined above. This timing, in principle agrees well with that found in the bean root by Howard and Pelc (1951).

It can be seen from Tables I and II and Fig. 1 that although DNA labelling with both isotopes gives essentially the same results, the values for adenine $^{14}$C are more consistent than those obtained with $^{32}$P. The reason for this difference is, firstly the differences between energy of the beta particles emitted by $^{14}$C and $^{32}$P. The higher energy $^{32}$P electrons result in a greater scatter and, consequently, in a higher background, thus interfering with the accuracy of the counts. Secondly, while the half life of $^{14}$C is a negligible problem when the isotope dilutions are added to the culture medium, variations in volume to allow for the decay are a source of error with $^{32}$P. Also, due to the decay of $^{32}$P and to the fact that $^{32}$P is supplied in $^{31}$P phosphate buffer, the ratio of $^{31}$ to $^{32}$P will not be constant : the degree of labelling of the phosphorus pool will be variable. These factors all contribute to the lesser accuracy of the counts in cultures with $^{32}$P labelling as compared with the $^{14}$C labelling.

FIG. 3.—The cell cycle related to DNA synthesis, measured by the incorporation of $^{32}$P or adenine $^{14}$C into DNA.

In some experiments with $^{32}$P orthophosphate a concentration of 10 $\mu$c./ml. medium was used instead of the usual 5 $\mu$c./ml. In short time cultures (3–6 hours) where the average grain count per nucleus is low, the higher isotope concentration results in an undesirable increase in the background. In cultures of 12 hours or over, however, the higher labelling of the pool increases the grain count per nucleus considerably and more reliable counts can be obtained than with the lower isotope concentration which labels some cells only slightly above the background of the long term cultures.

B. *Rate of DNA Synthesis in Different Types of Bone Marrow Cells.*

In these experiments only those types of cells were found to synthesise DNA which are known to be capable of mitosis. No differences were found between the same types of cells whether they originated in normal or leukaemic marrows.

The cell cycle as illustrated in Fig. 3 represents the average values for the majority of the dividing cells of the bone marrow, i.e., promyelocytes, myelocytes, pronormoblasts, basophilic normoblasts and early polychromatic normoblasts. In a few experiments, however, where it was found to be possible to count early normoblasts and early myeloid cells separately, a shorter cycle than in Fig. 3 was indicated for the normoblasts. Blast cells from acute leukaemia have shown a strikingly slower rate of DNA synthesis than the promyelocytes and myelocytes of the normal marrow or myelocytic leukaemia marrow (Table IV). No difference was found in this respect between normal or leukaemic serum as culture medium. This slow growth rate of the blast calls as opposed to the more mature forms was

TABLE IV.—*Comparison of the Rate of Adenine $^{14}C$ Uptake into DNA by Normal Bone Marrow Cells and Blast Cells from Leukaemia.*

| Type of marrow. | Culture in isotope (hr.). | % positive nuclei. | Av. grains/ nuclei. | Remarks. |
|---|---|---|---|---|
| 6 normal marrows | 0–6 | 21 (mean) | 15 | (Normal serum) |
| Blast cell leuk. No. 194 | 0–6 | 7 | 6 | ,, ,, |
| | | 5 | 5 | (leuk. serum) |
| Blast cell leuk. No. 183 | 0–6 | 1 | 4 | (Normal serum) |
| 5 normal marrows | 0–24 | 65 (mean) | $>30$ | ,, ,, |
| Blast cell leuk. No. 194 | 0–24 | 20 | $<25$ | ,, ,, |
| | | 20 | $<25$ | (leuk. serum) |

surprising in view of the rapid clinical course of the blast cell leukaemias: on the other hand it is compatible with the low mitotic index usually found in blast cell leukaemia marrows. Blast cells in normal marrows are too scanty to allow quantitative assessment of their cycle time, but the observations in the cultures suggested a similar cycle time to that obtained with leukaemic blast cells.

No appreciable DNA synthesis was detected in the lymphocytes whether normal or from lymphocytic leukaemia. Similarly, no appreciable DNA synthesis was detected in the plasmacytes from normal marrows. Plasmacytes from one case of multiple myeloma showed a limited amount of DNA synthesis, less than found in blast cells.

With increase in the degree of maturation the cells lose the ability to synthesise DNA. Thus no DNA synthesis was detected after the metamyelocyte stage, or the late polychromatic normoblast stage.

### C. *RNA Turnover.*

Smears not hydrolysed with N HCl, or even better, smears treated with the enzyme desoxyribonuclease give a picture of the ribose nucleic acid (RNA) turnover in the cells. It was found that the strongest uptake of RNA $^{32}P$ or RNA adenine $^{14}C$ is in the nucleus. The cytoplasm of the early normoblasts and promyelocytes has also shown uptake, but never to the same extent as the nucleus (Fig. 4–11). The very slight cytoplasmic uptake of $^{32}P$ or adenine $^{14}C$ into RNA of the plasma cells was surprising. Both plasma cells and lymphocytes show a good protein turnover as measured with methionine $^{35}S$ uptake. It seems that an active protein turnover is not necessarily accompanied by an active RNA turnover.

### D. *The Effect of X-ray Irradiation on DNA Synthesis*

Large doses of X-rays (5000 r in 15 minutes, 140 kV, l./mm. Al filter) exerted a marked inhibition on DNA synthesis. As is shown by Tables V and VI and Fig. 12, if in a 0–6 hour culture in isotope, radiation is delivered in the 3rd hour

TABLE V.—*Effect of 5000 r on the Incorporation of Adenine $^{14}C$ into DNA.*
(Radiosensitivity of the " S " period.)

| Exp. No. | Culture in isotope. | % positive nuclei. | Av. grains/nucleus. |
|---|---|---|---|
| 193 | 0–3 | 8 | 8 |
| 197 | 0–3 | 8 | 10 |
| 193 | 0–6 | 20 | >15 |
| 197 | 0–6 | 16 | >10 |
| 199 | 0–6 | 26 | 15 |
| 193 | 0X–6 | 1 | < 5 |
| 197 | 0X–6 | 1 | 5 |
| 193 | 0–3X–6 | 8 | <8 |
| 197 | 0–3X–6 | 5 | >5 |
| 199 | 0–3X–6 | 6 | <10 |

TABLE VI.—*Effect of 5000 r on the Incorporation of $^{32}P$ into DNA.*
(Radiosensitivity of the " S " period.)

| Exp. No. | Culture in isotope. | % positive nuclei. | Av. grains/nuclei. |
|---|---|---|---|
| 182 | 0–3 | 28 | — |
| 192 | 0–3 | 17 | 30 |
| 181 | 0–6 | 46 | — |
| 182 | 0–6 | 41 | — |
| 185 | 0–6 | 33 | — |
| 192 | 0–6 | 40 | >40 |
| 180 | 3–9 | 37 | — |
| *181 | 0X–6 | 14 | — |
| 192 | 0X–6 | 16 | 10 |
| 182 | 0–3X–6 | 26 | — |
| 185 | 0–3X–6 | 18 | — |
| 192 | 0–3X–6 | 33 | 30 |

((0–3X–6) then both the number of cells showing DNA autoradiographs and the grain count over the nuclei give similar counts to those in a 3 hour culture (0–3). This observation suggests that cells in the period of DNA synthesis are immediately and completely prevented from synthesising DNA. Radiation

FIG. 12.—Radiosensitivity of the " *S* " period. × = 5000r.

delivered at the beginning of the culture period in isotope (0X–6) also markedly inhibits DNA synthesis. The low grain counts and the low numbers of cells showing DNA autoradiograph suggested that it was probably the arrival of cells from the $G_1$ period into the $S$ period during the culture which gave the autoradiographs. However, if cells in the $G_1$ period had not been damaged by the radiation, then those at the end of the $G_1$ period at the time of irradiation should have entered the $S$ period and should have synthesised that amount of DNA normally synthesised in 6 hours. Since the grain counts indicated that subsequent to irradiation no cells, even with long culture times, synthesised more DNA than a 2–3 hour non-irradiated culture would (Fig. 12 and 13), it was suspected that the cells in the $G_1$ period suffered a latent damage.

Tables VII and VIII and Fig. 13 show that if the isotope was added to the cultures 3 or more hours after irradiation (X–3–24) hardly any DNA synthesis was observed, as opposed to those cultures in which the isotope was added imme-

TABLE VII.—*Effect of* 5000 *r on the Incorporation of* $^{14}C$ *Adenine into DNA*

(Demonstration of the " latent damage.")

| Exp. No. | Culture in isotope. | % positive nuclei. | Av. grains/nuclei. |
|---|---|---|---|
| 181 | 0–24 | 70 | — |
| 189 | 0–24 | 61 | — |
| 198 | 0–24 | 70 | 45 |
| 199 | 0–24 | 58 | >30 |
| 185 | 0–26 | 61 | — |
| 180 | 3–24 | 63 | — |
| 199 | 3–24 | 58 | >30 |
| 198 | 4–24 | 70 | 45 |
| 189 | 0X–24 | 12 | — |
| 198 | 0X–24 | 25 | 20 |
| 199 | 0X–24 | 20 | 15 |
| *180 | X–3–24 | 14 | — |
| 199 | X–3–24 | 10 | 6 |
| 198 | X–4–24 | 5 | 10 |

* Due to mistake in filtration the dose of irradiation in this culture was probably considerably less than 5000 r.

FIG. 13.—Demonstration of the latent damage. × = 5000 r.

TABLE VIII.—*Effect of 5000 r on the Incorporation of $^{32}P$ into DNA.*

(Demonstration of the " latent damage.")

| Exp. No. | Culture in isotope. | % positive nuclei. | Av. grains/nuclei. |
|---|---|---|---|
| 169 | 0–24 | 58 | — |
| 185 | 0–26 | 56 | — |
| 189 | 0–24 | 56 | — |
| 192 | 0–24 | 59 | >80 |
| 196 | 0–24 | 59 | 100 |
| 198 | 0–24 | 58 | >80 |
| 180 | 3–24 | 53 | — |
| 196 | 6–24 | 60 | >80 |
| 189 | 0X–24 | 30 | — |
| 192 | 0X–24 | 30 | 15 |
| 196 | 0X–24 | 21 | >20 |
| 196 | X–3–24 | 9 | <20 |
| 196 | X–6–24 | 7 | 20 |

diately following the irradiation (0X–24). These observations suggest that cells in the $G_1$ period receive a latent damage from a dose of 5000 r. The latent period is about 3 hours during which the cells may enter the $S$ period (if at the time of irradiation they were near the end of $G_1$ period). After about 3 hours, however, even these cells will be incapable of DNA synthesis.

The comparison of cultures incubated for 6 hours after irradiation (0X–6) and for 24 hours after irradiation (0X–24) indicated a certain amount of " post-irradiation recovery " (Fig. 14). The grain counts indicated, however, that this

FIG. 14.—Post-irradiation DNA synthesis.

was not a recovery in the sense of ability to synthesise normal amounts of DNA, but the entry of some $G_1$ cells into the $S$ period for a limited time. It appears that all $G_1$ cells are damaged by a dose of 5000 r.

The effect of irradiation on the $G_2$ period is indicated by the fact that no mitoses were observed in any of the irradiated cultures.

A dose of 5000 r produced a marked degeneration of the cells in the cultures, but over 6 hour culture times were needed to show the first morphological signs of post-irradiation degeneration. The complete inhibition of mitoses was, however, immediate.

In agreement with previous findings by other workers it was found that even this large dose of radiation produced only a moderate decrease in RNA turnover. This should be compared with the effect of barbiturate (Nembutal Abbot 0·5 mg./ml. medium) which not only markedly inhibited DNA synthesis, but also markedly inhibited RNA turnover in the cells (Table IX).

TABLE IX.—*Effect on Nembutal on Adenine* $^{14}C$ *Uptake.*

| Type in culture. | Culture in isotope. (hr.). | DNA %+ nuclei. | DNA Av. grs./nuclei. | RNA %+ nuclei. | RNA Av. grs./nuclei. |
|---|---|---|---|---|---|
| Control | 0–24 | 70 | 45 | 90 | 80 |
| Nembutal 0·5 mg./l. | 0–24 | 11 | 10 | 90 | 20 |

### DISCUSSION.

DNA synthesis was defined above as the incorporation of $^{32}P$ or adenine $^{14}C$ into DNA. In a sense this could be better defined as the assembly of the DNA molecule. Experiments with the synthesis of the purines and pyrimidines from labelled one carbon compound precursors (*e.g.*, formate $^{14}C$) are in progress.

The timing of the cell cycle is, in principle, in good agreement with that described in the bean root cell by Howard and Pelc (1951). It is expected that the timing of the cell cycle in different mammalian tissues will yield different lengths of cycles. It remains to be seen whether in longer or shorter cycles than illustrated above, all periods are longer or shorter, or only $G_1$ (and possibly $G_2$). It would not be surprising to find that DNA synthesis is such a rigidly standardised process in Nature that the synthesis of a given diploid amount always takes the same time.

The large dose of radiation employed in these experiments was deliberately chosen. Experiments with lower doses and with varying dose rates are in progress, and it may be that the response of the cells under such conditions will be qualitatively as well as quantitatively different from those described in this paper.

This work is part of an investigation carried out under a full grant from the British Empire Cancer Campaign.

### SUMMARY.

(1) Using the incorporation of $^{32}P$ and adenine $^{14}C$ into DNA as indicators of DNA synthesis it was possible to study and to time the period of DNA synthesis in the intermitotic cycle of human bone marrow cells *in vitro*. The total cycle time was found to be of the order of 40–48 hours for the average dividing bone marrow cells (promyelocytes, myelocytes, pronormoblasts, basophilic and polychromatic normoblasts). The period of DNA synthesis was found to be of the order of 12–15 hours taking place in the second half of the cycle, and divided from the mitosis by a 3–4 hours non-synthesising period.

(2) Only cell types capable of division showed DNA synthesis.

(3) No difference was found between the same types of cells whether originating in normal marrows or in marrows of patients with leukaemia.

(4) Blast cells from acute leukaemias showed a markedly slower rate of DNA synthesis (growth rate) than promyelocytes and myelocytes from normal or leukaemic marrows.

(5) The early nucleated red cell series (pronormoblasts and basophilic normoblasts) showed a slightly faster rate of DNA synthesis than the promyelocytes and myelocytes.

(6) No appreciable DNA synthesis was observed in the lymphocytes whether normal or leukaemic.

(7) 5000 r X-ray irradiation inhibited DNA synthesis immediately and completely.

(8) Cells before the period of DNA synthesis developed a latent damage after 5000 r which either completely abolished or considerably decreased their ability to synthesise DNA.

### REFERENCES.

Davidson, J. N., Leslie, J., and White, J. C.—(1951*a*) *J. Path. Bact.*, **63**, 471.—(1951*b*) *Lancet*, i, 1287.

Hamilton, L.—(1953) *Nature*, **172**, 457.

Howard, A., and Pelc, S. R.—(1951) Paper in 'Isotopes in Biochemistry.' Ciba Foundation. London (Churchill).

Lajtha, L. G.—(1952*a*) *J. clin. Path.*, **5**, 67.—(1952*b*) *Exp. Cell Res.*, **3**, 696.—(1954*a*) *J. photogr. Sci.* (in press).—(1954*b*) *Nature*, **173**, 587.

Lamerton, L. F.—(1950) *cit. Pelc. S.R.* in 'Isotopes in Biochemistry.' Ciba Foundation. London (Churchill).